# Prevalence of Flp Pili-Encoding Plasmids in *Cutibacterium acnes* Isolates Obtained from Prostatic Tissue

**DOI:** 10.3389/fmicb.2017.02241

**Published:** 2017-11-16

**Authors:** Sabina Davidsson, Jessica Carlsson, Paula Mölling, Natyra Gashi, Ove Andrén, Swen-Olof Andersson, Elzbieta Brzuszkiewicz, Anja Poehlein, Munir A. Al-Zeer, Volker Brinkmann, Carsten Scavenius, Seven Nazipi, Bo Söderquist, Holger Brüggemann

**Affiliations:** ^1^Department of Urology, Faculty of Medicine and Health, Örebro University, Örebro, Sweden; ^2^Department of Laboratory Medicine, Clinical Microbiology, Faculty of Medicine and Health, Örebro University, Örebro, Sweden; ^3^Department of Genomic and Applied Microbiology, Institute of Microbiology and Genetics, University of Göttingen, Göttingen, Germany; ^4^Department of Molecular Biology, Max Planck Institute for Infection Biology, Berlin, Germany; ^5^Microscopy Core Facility, Max Planck Institute for Infection Biology, Berlin, Germany; ^6^Department of Molecular Biology and Genetics, Aarhus University, Aarhus, Denmark; ^7^Department of Biomedicine, Aarhus University, Aarhus, Denmark

**Keywords:** *Cutibacterium acnes*, *Propionibacterium acnes*, plasmid, fimbrial low-molecular weight protein, pili, tight adherence, prostate cancer

## Abstract

Inflammation is one of the hallmarks of prostate cancer. The origin of inflammation is unknown, but microbial infections are suspected to play a role. In previous studies, the Gram-positive, low virulent bacterium *Cutibacterium* (formerly *Propionibacterium*) *acnes* was frequently isolated from prostatic tissue. It is unclear if the presence of the bacterium represents a true infection or a contamination. Here we investigated *Cutibacterium acnes* type II, also called subspecies *defendens*, which is the most prevalent type among prostatic *C. acnes* isolates. Genome sequencing of type II isolates identified large plasmids in several genomes. The plasmids are highly similar to previously identified linear plasmids of type I *C. acnes* strains associated with acne vulgaris. A PCR-based analysis revealed that 28.4% (21 out of 74) of all type II strains isolated from cancerous prostates carry a plasmid. The plasmid shows signatures for conjugative transfer. In addition, it contains a gene locus for tight adherence (*tad*) that is predicted to encode adhesive Flp (fimbrial low-molecular weight protein) pili. In subsequent experiments a *tad* locus-encoded putative pilin subunit was identified in the surface-exposed protein fraction of plasmid-positive *C. acnes* type II strains by mass spectrometry, indicating that the *tad* locus is functional. Additional plasmid-encoded proteins were detected in the secreted protein fraction, including two signal peptide-harboring proteins; the corresponding genes are specific for type II *C. acnes*, thus lacking from plasmid-positive type I *C. acnes* strains. Further support for the presence of Flp pili in *C. acnes* type II was provided by electron microscopy, revealing cell appendages in *tad* locus-positive strains. Our study provides new insight in the most prevalent prostatic subspecies of *C. acnes*, subsp. *defendens*, and indicates the existence of Flp pili in plasmid-positive strains. Such pili may support colonization and persistent infection of human prostates by *C. acnes*.

## Introduction

Prostate cancer is the most common cancer among men (Global Burden of Disease Cancer Collaboration et al., 2017). Despite the high incidence the etiology is still largely unknown. Accumulating evidence has, however, highlighting the role of chronic inflammation in prostate carcinogenesis; repeated tissue damage and regeneration, in the presence of high levels of cytokines, chemokines and reactive oxygen species, could initiate inflammation-induced prostate cancer (De Marzo et al., 2007). Both chronic and acute inflammation is frequently observed in histological examinations of prostate specimens. Since this inflammation is present throughout the entire gland and not exclusively at the tumor site, an infectious etiology of the inflammation has been suggested and therefore infection-induced inflammation may have a role in prostate cancer development (Sfanos and De Marzo, 2012; Gurel et al., 2014).

*Propionibacterium acnes* is a facultative anaerobe, Gram-positive bacterium that constitutes part of the skin microbiota. It was recently renamed to *Cutibacterium acnes* (Scholz and Kilian, 2016). In addition to the prevalence on the skin, *C. acnes* is also present at other body sites such as the oral cavity, intestinal tract, and conjunctiva. Although generally regarded as a commensal, *C. acnes* has been associated with skin disorders such as acne vulgaris (Beylot et al., 2014). It is also associated with a number of postoperative and medical device-related infections, including infections of the shoulder after arthroplasty, sternal wound infections after cardiothoracic surgery as well as with other conditions such as sarcoidosis, dental infections and recently intervertebral disk degeneration (Perry and Lambert, 2011; Achermann et al., 2014; Brüggemann, 2016). In recent reports *C. acnes* has also been frequently detected in prostate tissue from men with benign prostatic hyperplasia and prostate cancer (Cohen et al., 2005; Alexeyev et al., 2007; Sfanos et al., 2008; Fassi Fehri et al., 2011; Mak et al., 2013; Bae et al., 2014; Davidsson et al., 2016). Supporting evidence to the hypothesis that *C. acnes* is a contributing factor in infection-induced prostate cancer was recently presented by our group showing that *C. acnes* was significantly more common in prostate tissue samples obtained from men with prostate cancer compared to prostate tissue with no histological evidence of the disease (Davidsson et al., 2016).

The contradiction between the lifestyle as a harmless commensal on the skin and an opportunistic pathogen may be partly explained by strain-specific properties. *C. acnes* strains can be categorized in the main phylotypes IA, IB, IC, II, and III according to several typing schemes including SLST/MLST (McDowell et al., 2005, 2011, 2012; Lomholt and Kilian, 2010; Kilian et al., 2012; Scholz et al., 2014). Type IA strains are predominantly found on the skin of the face and upper body (Petersen et al., 2017) and certain CC of type IA strains are also associated with acne vulgaris; in contrast, type IB and II strains are associated with deep tissue infections (Lomholt and Kilian, 2010; McDowell et al., 2012, 2013; Fitz-Gibbon et al., 2013). The dual role of *C. acnes* as commensal and opportunistic pathogen has raised the question if there are specific *C. acnes* strains colonizing the prostate with elevated pathogenic potential and therefore more prone to induce inflammation and subsequently cellular transformation. We recently found that type II, now reclassified as *C. acnes* (*P. acnes*) subsp. *defendens* (McDowell et al., 2016), was the most abundant type among strains in prostatic tissue (Davidsson et al., 2016).

Here, we further analyzed prostatic type II strains by genome sequencing. The sequencing results revealed the presence of a large plasmid in a substantial number of type II isolates. The plasmid, highly similar to previously found linear plasmids in type IA isolates associated with acne vulgaris, harbors a *tad* locus that is predicted to encode the production of Flp (fimbrial low-molecular weight protein) pili. Here, we identified differences between the plasmids from type IA and type II strains and could detect plasmid-encoded factors in the secreted and surface-exposed protein fractions of type II strains, including a putative pilin subunit. Further analyses revealed the presence of cell appendages, resembling Flp pili, in *tad*-positive *C. acnes* subsp. *defendens* strains.

## Materials and Methods

### Bacterial Isolates, Cultivation and Strain Typing

*Cutibacterium acnes* isolates from prostatic tissue were previously obtained from men who underwent radical prostatectomy or cystoprostatectomy, with or without histological findings for prostate cancer (Davidsson et al., 2016). In total, 181 *C. acnes* prostatic isolates were used in the present study:

– 134 isolates from men (*n* = 100) diagnosed with prostate cancer.
– 29 isolates from men (*n* = 37) diagnosed with bladder cancer with additional histological findings for prostate cancer.
– 18 isolates of men (*n* = 50) diagnosed with bladder cancer without any histological findings for prostate cancer.

The tissue collection protocol and sampling processing have been described in detail previously (Davidsson et al., 2016). Briefly, a biopsy gun and sterile, single-use biopsy needles were used to obtain six tissue cores from each patient’s prostate gland. All biopsies were performed at the operating theater and immediately transported to the Department of Laboratory Medicine, Clinical Microbiology, at the Örebro University Hospital for cultivation and characterization of *C. acnes*.

Initial *C. acnes* typing was done by amplifying and sequencing the *tly* gene as previously described to classify the strains into type IA, IB, II, or III (Davidsson et al., 2016). For all genome-sequenced strains in this study the MLST and SLST types were determined according to the schemes of Kilian et al. (2012) and Scholz et al. (2014), respectively. All type II sequence types of the SLST scheme are deposited at http://medbac.dk/slst/pacnes.

The *Clostridium leptum* strain DSM753 (ATCC 29065) was obtained from the Leibniz Institute DSMZ-German Collection of Microorganisms and cell cultures, Braunschweig, Germany.

All strains were grown in RCM under anaerobic conditions.

### Real-Time PCR Conditions and Sequencing

For crude DNA extraction, bacteria were harvested with a 10 μl sterile loop (ca. 5 μl pellet volume) from an agar plate and resuspended in 1 ml sterile water (OD_600 nm_ = ca. 8). The suspension was incubated at 90°C for 45 min, and then centrifuged at 5,000 *g* for 15 min. The resulting supernatant containing the genomic DNA was stored at -20°C prior to PCR analysis.

To investigate the presence of a large plasmid, a fragment of the gene *tadA* gene was amplified with primers *tadA-*F (5′-GGTGGGTAACCATGAGGTGG) and *tadA-*R (3′-GATCGCGTGGATACGGAACT) to generate a 196 bp amplicon. The PCR including melting curve analysis was performed in the CFX96 Touch TM real-time PCR detection system (Bio RAD, Sundbyberg, Sweden). Each reaction mixture (25 μl) contained: 1X iTaq Universal SYBR Green (Bio-Rad), 0.5 μM of each primer and 4 μl DNA template. The thermal cycling conditions included an initial pre-incubation at 95°C for 10 min followed by 40 cycles of 95°C for 15 s, 60°C for 60 s, and 65°C for 5 s.

Prior to sequencing, the PCR products were purified using MultiScreen PCRμ96 plate (Millipore, Molsheim, France), according to the manufacturer’s instructions. One μl of the purified PCR product was then cycle-sequenced using Big Dye Terminator v 3.1 according to the manufacturer’s instructions (Thermo Fisher Scientific). The cycle-sequencing PCR consisted of 25 cycles of 96°C for 10 s, 60°C for 5 s, and 60°C for 4 min. Subsequently, the reactions were purified using ethanol-sodium acetate precipitation. The nucleotide sequences were determined by capillary electrophoresis using a 3500 genetic analyzer (Thermo Fisher Scientific). The sequences were aligned and compared to the *tadA* gene of the plasmid in strain 15.1.R1 (locus tag: AFH37449).

### Genome Sequencing of *C. acnes* Prostate Isolates and *Cl. leptum* DSM 753

Genomic DNA from 17 *C*. *acnes* strains and the *Cl. leptum* strain was isolated using the MasterPure Gram-positive DNA Purification Kit (EpiCentre MGP04100) according to the manufacturer’s instructions. The purity and quality of the gDNA were assessed on a 1% agarose gel and with a nanodrop apparatus (Thermo Fisher Scientific). Extracted DNA was used to prepare Nextera XT shotgun libraries for the Genome Analyzer II (Illumina, San Diego, CA, United States) with a 112-bp paired-end sequencing run. Libraries were prepared according to the manufacturer protocol at the Göttingen Genomics Laboratory, Germany. Raw reads were quality controlled with FastQC v0.11.2^1^ and subsequently trimmed using Trimmomatic 0.32^2^ to remove sequences with quality scores lower than 20 (Illumina 1.9 encoding) and remaining adaptor sequences. *De novo* assembly was done using the SPAdes v3.5 software (Bankevich et al., 2012). Automated genome annotation was carried out using RAST (Aziz et al., 2008). In addition, manual annotations which BLAST and PFAM was done for the plasmid genes. The GenBank accession numbers of the draft genome sequences are: LKVB00000000 (strain 09-9), LKVC00000000 (09-23), MVCB00000000 (09-323), MVCC00000000 (12-89), MVCD00000000 (10-482), MVCE00000000 (11-78), MVCF00000000 (11-88), MVCG00000000 (11-90), MVCH00000000 (10-43), MVCI00000000 (09-109), MVCJ00000000 (09-263), MVCK00000000 (10-113), MVCL00000000 (10-118), MVCM00000000 (10-167), MVCN00000000 (11-49), MVCO00000000 (11-79), MVCP00000000 (11-356). For *Cl. leptum* DSM 753 the GenBank accession number is NOXF00000000.

### Bioinformatics and Comparative and Phylogenetic Analyses

For comparative plasmid analysis, the programs ACT^3^ and BRIG (Alikhan et al., 2011) were used. In addition to the nine plasmids identified here, three previously published *tad* locus-containing plasmids of *C. acnes* were used: p15.1.R1 (accession number: JQ612072), pIMPLE-HL096PA1 (CP003294) and the plasmid contig in strain 523 (JVDS01000001). Sequence comparison of the plasmids was done with MAFFT^4^. Phylogenetic trees were built in Mega v6 (Tamura et al., 2013). For phylogenomic analysis of the genome sequences the program CSI Phylogeny (v1.4) was used^5^. Here the genome of strain 09-9 was used as reference; single nucleotide polymorphisms (SNPs) were called in the other type II genomes and phylogeny was inferred based on the concatenated alignment of the SNPs. For CRSIPR/cas analysis the CRISPR-finder tool was used^6^. For comparison of *Cl. leptum* genomes, the previously sequenced genome of strain DSM 753 with the GenBank accession number ABCB02000000 was used.

### Scanning Electron Microscopy

*Cutibacterium acnes* was grown in RCM under anaerobic condition to on OD_600 nm_ of approximately 1. Bacterial cells were fixed with 2.5% glutaraldehyde, post-fixed using repeated incubations with 1% osmium tetroxide/1% tannic acid, dehydrated with a graded ethanol series, critical point dried and coated with 3 nm platinum/carbon. Specimens were analyzed in a Leo 1550 scanning electron microscope.

### Enrichment of Secreted and Surface-Attached Proteins of *C. acnes*

For the collection of extracellular, secreted proteins, *C. acnes* strains were grown in RCM to late exponential phase (OD_600 nm_ approximately 1). The cultures were centrifuged for 30 min at 4,000 *g* and 4°C. Supernatant was filtered through a 0.22-μm-pore-size membrane filter to remove residual bacteria. Extracellular proteins were precipitated using a modified trichloroacetic acid (TCA) method (Komoriya et al., 1999). In brief, the supernatant filtrate was mixed with TCA to a final concentration of 10% and incubated overnight at 4°C on a tube rotator. The mixture was centrifuged for 20 min (20,000 *g* and 4°C) and the resulting pellet was resuspended in 1 ml of ice-cold acetone, transferred to Eppendorf tubes and submerged into an ultrasonic bath for 10 min. The resuspended pellet was washed twice with acetone and the resulting pellet was air-dried and stored at -80°C.

In order to collect also surface-attached appendages, such as pili, we used a pili-shearing protocol (Laurenceau et al., 2013). In brief, *C. acnes* strains were grown in RCM to late exponential phase (OD_600 nm_ approximately 1) and bacterial cell were harvested by low centrifugation (2,500 *g*, 3 min); the pellet was suspended in 1 ml broth and vortexed vigorously for 1 min to apply mechanical force. The suspension was centrifuged twice (13,000 *g*, 5 min) to separate bacterial cells from the appendages-enriched supernatant. The supernatant was then precipitated with TCA as described above.

### Mass Spectrometry

Proteins in the secreted and sheared fractions were identified with MS. Nano-electrospray ionization MS/MS (nanoESI-MS/MS) analyses were performed on an EASY-nLC II system (Thermo Fisher Scientific) connected to a TripleTOF 5600+ mass spectrometer (AB SCIEX) operated under Analyst TF 1.6.1 control. The trypsin-digested samples were suspended in 0.1% formic acid, injected, trapped and desalted on a precolumn. The peptides were eluted and separated on a 15 cm analytical column (75 μm i.d.), pulled in-house (P2000 laser puller, Sutter Instrument). Trap and analytical column were packed with ReproSil-Pur C18-AQ 3 μm resin (Dr. Maisch GmbH). Peptides were eluted from the analytical column at a flow rate of 250 nl/min using a 30 min gradient from 5 to 35% of solution B (0.1% formic acid, 100% acetonitrile). The collected MS files were converted to Mascot generic format (MGF) using the AB SCIEX MS Data Converter beta 1.1 (AB SCIEX) and the “protein pilot MGF” parameters. The generated peak lists were searched using an in-house Mascot search engine (Matrix Science) against all *C. acnes* proteins in the UniProt database as well as against all *tad* plasmid-encoded proteins (the plasmid p09-9 was used). Search parameters were allowing one missed trypsin cleavage site and propionamide as a fixed modification with peptide tolerance and MS/MS tolerance set to 10 ppm and 0.2 Da, respectively.

## Results

### Collection of *C. acnes* Prostate Isolates

One hundred and eighty-one *C. acnes* isolates were used in this study, previously collected from prostatic tissue. The details of this strain collection as well as the prostatic issue collection protocol and sampling procedure have been described previously (Davidsson et al., 2016). In brief, the strain collection included 134 *C. acnes* isolates obtained from cancerous prostates of 100 men diagnosed with prostate cancer who underwent radical prostatectomy. In addition, *C. acnes* isolates were included from prostates of 87 patients who were diagnosed with bladder cancer and underwent cystoprostatectomy. The removed prostates of these patients were histologically investigated. Fifty prostates had no histological evidence for prostate cancer; here, 18 *C. acnes* isolates were obtained. Thirty-seven prostates of bladder cancer patients showed histological evidence for prostate cancer; here, 29 *C. acnes* isolates were obtained.

Previously undertaken *tly* gene-based phylotyping showed that 53 (29.3%) *C. acnes* isolates are type IA, 34 (18.8%) type IB, 84 (46.4%) type II, and 10 (5.5%) type III (Davidsson et al., 2016). The predominance of type II strains among prostatic *C. acnes* isolates prompted us to get further insight by means of genome sequencing of type II strains isolated from cancerous and healthy prostates in order to evaluate if type II strains might possess specific properties enabling them to colonize prostatic tissue.

### Presence of Large Plasmids in Prostatic *C. acnes* Isolates

First, two type II strains were randomly selected, strain 09-9 isolated from a prostate cancer case and strain 09-23 from a healthy prostate. Genome sequence assembly resulted in 16 and 12 contigs for strains 09-9 and 09-23, respectively. Phylogenetic analyses showed that both strains belong to the MLST type CC53 and the SLST type K1, according to the Aarhus typing scheme (Kilian et al., 2012; Scholz et al., 2014). However, the genome size differed between the two strains: specific to strain 09-9 was a 53 kb large contig that shared high similarity with previously described linear plasmids of *C. acnes*, namely in strains 15.1.R1 and HL096PA1 (Brüggemann et al., 2012; Kasimatis et al., 2013). These strains belong to phylotype IA and accordingly, previous studies identified plasmids almost exclusively in type IA strains (Tomida et al., 2013; Scholz et al., 2016). Like the plasmids in type IA strains, the newly identified plasmid in the type II strain 09-9 also encoded a *tad* locus that is predicted to be involved in adhesion and tissue colonization.

These results prompted us to test for the presence of *tad*-locus containing plasmids in all 181 prostatic *C. acnes* isolates by a PCR-based approach. A region within the *tad* locus was identified (*tadA*) that is conserved among the three plasmids of strains 15.1.R1, HL096PA1 and 09-9. A *tadA*-positive PCR was obtained in 20% (37 of 181) of all strains (**Table 1**). The distribution of *tadA*-positive strains among the different *C. acnes* types varied between 15% (type IA) and 26% (type II). No *tadA*-positive type III strain (*C. acnes* subsp. *elongatum*) was present in the strain collection. These results indicate that the *tad* locus-containing plasmid is present in a substantial number of strains isolated from the prostate, and the plasmid was most often found in type II strains.

**Table 1:** Amplification of *tadA*, indicative of the presence of a large plasmid in prostatic *C. acnes* isolates.

	*tadA* +	*tadA* -	Total number
All types	37 (20)	144 (80)	181
Type IA	8 (15)	45 (85)	53
Type IB	7 (21)	27 (79)	34
Type II	22 (26)	62 (74)	84
Type III	0 (0)	10 (100)	10

*(in brackets: percentage).*

Further analysis of the prevalence of the plasmid in strains isolated from men with prostate cancer revealed that 10.4% (5/48) of type IA strains, 19.4% (6/31) of type IB strains and 28.4% (21/74) type II strains were *tadA*-positive (**Table 2**). The plasmid was also present in one type II strain (out of 10) obtained from a cancer-free prostate. Due to the low number of *C. acnes* isolates obtained from healthy prostates a meaningful comparison of the plasmid prevalence in strains obtained from men with and without prostate cancer was not possible.

**Table 2:** Distribution of *tadA*-positive (+) and -negative (**-**) *C. acnes* strains among isolates obtained from prostate cancer (PCa) cases and controls.

	Type IA		Type IB		Type II	
	+	-	+	-	+	-
PCa cases	4	33	3	16	21	47
Controls	3	2	1	2	1	9
Controls with PCa	1	10	3	9	0	6

### Genome Sequencing Analysis of *C. acnes* Prostate Isolates

Next, we wanted to analyze plasmid-positive prostate isolates in more detail in order to investigate if the plasmids are conserved among different *C. acnes* types. Based on the *tadA*-specific PCR, 17 strains were genome-sequenced, comprising six type IA, two type IB and nine type II strains. These included 11 *tadA*-positive and six *tadA*-negative strains with identical SLST types (**Table 3**). In agreement with the *tadA* PCR, 11 out of 17 strains contained sequence contigs that were highly similar to the known plasmids from strains 15.1.R1 (the plasmid is hereafter called p15.1.R1) and HL096PA1 (plasmid pIMPLE-HL096PA1) (Brüggemann et al., 2012; Kasimatis et al., 2013). In order to get complete plasmid sequences without remaining sequence gaps, we extracted for each strain the contigs with homology to p15.1.R1, and closed the remaining gaps by context-specific gap closure PCRs. This resulted in a total of nine complete plasmids, obtained from two type IA strains, one type IB strain and six type II strains (**Table 3**). The GC content of these plasmids varied between 61.7 and 63.0% thus is higher than the GC content of the chromosome (in average 60.0% for type I and 60.1% for type II).

**Table 3:** Features of sequenced *C. acnes* isolates and their large plasmids.

Strain	Source	Phylo-type	SLST type	Genome size (kb)	Contigs	Plasmid size (bp)	Plasmid GC content (%)	CRISPR/cas (# spacers)
12-89	Healthy	IA1	A1	2480	9	–	–	No
11-78	Tumor	IA1	A1	2528	15	54033	62.4	No
09-263	Tumor	IA1	A1	2484	14	–	–	No
10-113	Healthy	IA1	C5	2527	15	47433	62.9	No
10-167	Tumor	IA1	A1	2525	11	few gaps	62.5	No
10-118	Healthy	IA1	A1	2475	13	–	–	No
11-88	Tumor	IB	H1	2545	14	–	–	No
11-90	Tumor	IB	H1	2597	15	53358	62.4	No
09-9	Tumor	II	K1	2532	16	53137	63.0	Yes (1)
09-23	Healthy	II	K1	2476	12	–	–	Yes (3)
09-323	Tumor	II	K8	2537	11	53582	62.9	Yes (2)
10-482	Healthy	II	K8	2541	12	57860	61.7	Yes (1)
10-43	Tumor	II	K2	2565	79	56106	62.7	Yes (6)
11-79	Tumor	II	K2	2539	8	54149	62.9	Yes (2)
11-356	Tumor	II	K2	2521	36	–	–	Yes (2)
09-109	Tumor	II	K5	2538	11	53516	63.0	Yes (1)
11-49	Tumor	II	K1	2540	17	Few gaps	63.0	Yes (3)

### Comparative Analysis of Plasmids from Type I and Type II Strains

**FIGURE 1:**
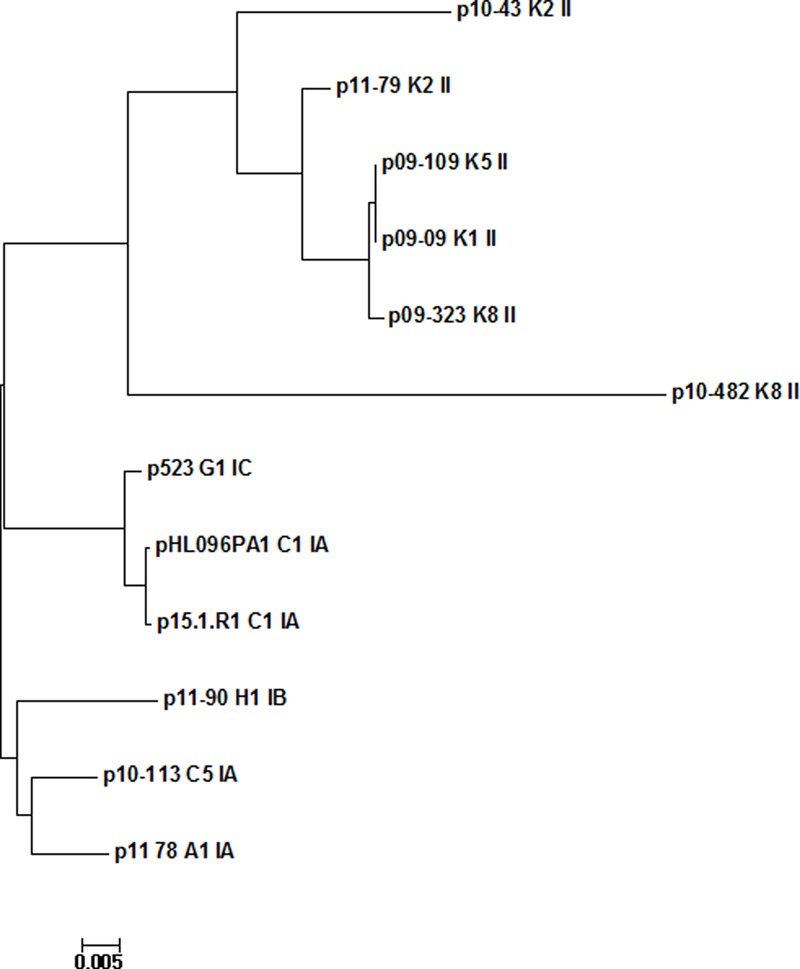
Phylogenetic analysis of large plasmids from *C. acnes* types I and II strains. Plasmids of type I strains [SLST types A1, C1, C5 (all type IA), G1 (type IC), H1 (type IB)] cluster together in two clades and are distinct from a clade of plasmids from type II strains (SLST types K1, K2, K5, K8). Sequences were aligned with MAFFT and the phylogenetic tree was built in Mega.

**FIGURE 2:**
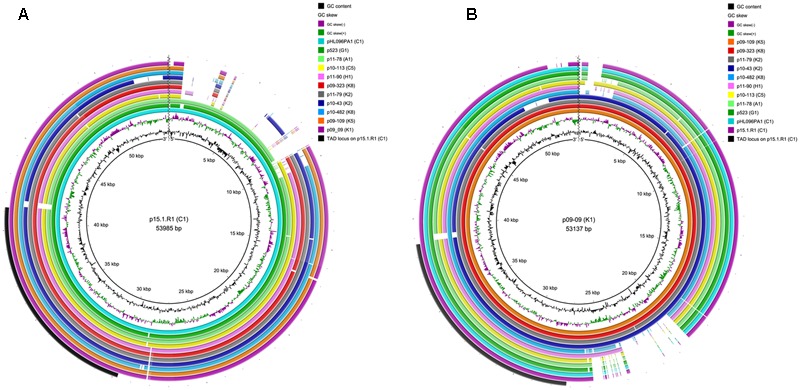
Comparison of 12 large plasmids of *C. acnes* type I and II strains. **(A)** The plasmid of the type IA strain 15.1.R1 was taken as the reference sequence and compared to 11 fully sequenced plasmids; **(B)** the plasmid of the type II strain 09-9 was taken as reference. Type-specific plasmid regions can be detected, e.g., a type IA/IC-specific region at the 5′-end of the plasmid and a type II-specific region in close vicinity to the *tad* locus. Note the conservation of the *tad* lcous in all plasmids. The maps were created with BRIG. The jagged line was introduced to illustrate that the plasmids are linear and not circular.

We compared all existing plasmids, i.e., the nine newly obtained plasmids and the previously available ones. Included in the comparison was another completed plasmid that was found in database searches, present in strain 523, a type IC isolate. The phylogenetic analysis of the 12 plasmids showed that plasmid sequences clustered according to the phylogeny of their host strains, i.e., the six plasmids of type I strains clustered in two clades and differed from a clade of six plasmids from type II strains (**Figure 1**); p10-482, a plasmid from a K8 strain, seems to be an outlier. A more detailed plasmid analysis was carried out using the comparison tools BRIG and ACT. The analyses further highlighted cluster-specific plasmid regions (**Figure 2**). Plasmids from type I strains contained a specific region at the 5′-end of the plasmid that is absent in plasmids from type II strains (**Figure 2A**); this region contains 17 genes, most of them encoding proteins with unknown functions. Two genes encode for proteins that have ParA domains predicted to be involved in chromosome/plasmid segregation/partition. One gene encodes a mRNA-degrading endonuclease similar to RelE, a toxin component of the RelBE type II toxin-antitoxin system.

**FIGURE 3:**
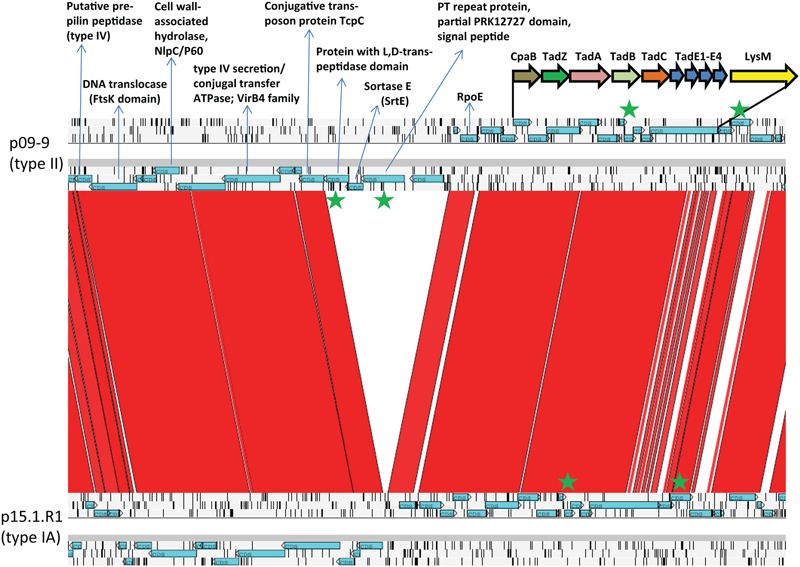
Plasmid loci encoding possible conjugative and host-interacting functions in *C. acnes* and their detection in cell surface and secreted fractions. Present on all plasmids is a *tad* locus encoding the biosynthesis of Flp/type IVb pili. In addition, all plasmids encode functions related to their conjugative transfer. A type II-specific locus was identified that encodes secreted or surface-exposed proteins. The comparison of the loci of p09-9 and p15.1.R1 and was done with ACT; regions of high similarity between the two plasmids is represented in red. Highlighted (green stars) are the genes whose proteins were detected by mass spectrometry in secreted and surface-exposed cell fractions.

All plasmids possess the previously described *tad* locus (Brüggemann et al., 2012; Kasimatis et al., 2013); the locus on plasmids from types I and II strains is highly similar. However, and interestingly, there is an insertion upstream of the *tad* locus in type II plasmids (**Figure 2B**). This insertion contains four genes; two of them encode proteins with signal peptides, indicative of their export (**Figure 3**). One protein (APS60_12543) contains PT repeats that are also found in a few other surface-exposed *C. acnes* proteins, including the dermatan sulfate-binding adhesin DsA1 that has recently been characterized as fibrinogen-binding protein (Grange et al., 2017). The adjacent gene encodes a sortase of the SrtE family. Sortases are important for the anchorage of surface proteins that contain a C- terminal sorting signal (e.g., LPXTG- or LAXTG-motifs).

### Proteomic Investigation of the Surface-Attached and Secreted Proteins of *C. acnes* subsp. *defendens*

Next, we wanted to investigate if the *tad* locus is functional. This gene cluster, present in several Gram-positive as well as Gram-negative bacterial species, encodes a secretion system for the assembly of adhesive Flp (fimbrial low-molecular-weight protein) pili, also called type IVb pili or Tad pili (Tomich et al., 2007). Pilin subunits of Flp pili have been identified, forming a unique subclass within the type IVb prepilin family (Imam et al., 2011). Our search for pilin subunits identified four possible candidates (TadE1-TadE4, APS60_12595 to APS60_12610 in strain 09-9) that are encoded in a cluster downstream of *tadC* (**Figure 3**). TadE1 (APS60_12595) contains a motif (G/SSTVET) that is similar to the predicted prepilin peptidase cleavage site (G/XXXXEY) (Tomich et al., 2006; Angelov et al., 2015), and TadE2, TadE3 and TadE4 share a similar N-terminus that does not represent a signal peptide; these three proteins have some similarity with the pseudopilin TadE, e.g., TadE2 (APS60_12600) contains the TadE/pfam07811 domain (**Supplementary Figures S1A,B**).

To detect the presence of pili in plasmid-positive *C. acnes* we first analyzed the bacterial surface-attached and secreted proteomes by gel-free MS. Bacteria were grown under anaerobic conditions to the post-exponential growth phase. Besides the harvest and identification of secreted proteins in bacterial culture supernatants, we used a mechanical shearing protocol in order to enrich surface-exposed factors including pili prior to MS identification. The proteomic analyses could reveal the presence of four plasmid-encoded proteins, including TadE2 (ASP60_12600), in both, secreted and sheared fractions of plasmid-positive *C. acnes* strains, but not in plasmid-negative strains (**Table 4, Supplementary Figure S1C** and Table S1). These results show that the plasmid is indeed functional; it is likely that Flp pili are formed in plasmid-positive type II *C. acnes* strains. Interestingly, two other identified plasmid-borne proteins are encoded in a type II-specific insertion just upstream of the *tad* locus, including the DsA1-like factor (APS60_12543) with repetitive motifs mentioned above (**Table 4** and **Figure 3**).

**Table 4:** Identification of plasmid-encoded proteins in secreted and surface sheared fractions of two *C. acnes* strains.

Strain	Fraction	Locus ID (in p09-9)	Annotation	MW	Score	Significant matches	Coverage (%)	Unique peptides
11-79	Secretome	APS60_12543	PT repeat protein, partial FlhF domain (type II specific)	62.5	577	15	20.8	11
11-79	Secretome	APS60_12625	Hypothetical protein, signal peptide	30.7	520	12	27	6
11-79	Secretome	APS60_12535	L,D-peptidoglycan transpeptidase domain protein (type II specific)	34.5	200	6	20.5	5
11-79	Secretome	APS60_12600	TadE family protein (TadE2)	12.9	119	2	26.4	2
11-79	Sheared	APS60_12625	Hypothetical protein, signal peptide	30.7	1009	19	39.2	8
11-79	Sheared	APS60_12543	PT repeat protein, partial FlhF domain (type II specific)	62.5	823	22	19.6	12
11-79	Sheared	APS60_12600	TadE family protein (TadE2)	12.9	242	4	26.4	2
11-79	Sheared	APS60_12535	L,D-peptidoglycan transpeptidase domain protein (type II specific)	34.5	145	6	14.4	4
09-9	Secretome	APS60_12625	Hypothetical protein, signal peptide	30.7	455	12	27	6
09-9	Secretome	APS60_12600	TadE family protein (TadE2)	12.9	213	4	44.8	3
09-9	Sheared	APS60_12625	Hypothetical protein, signal peptide	30.7	895	21	47.8	9
09-9	Sheared	APS60_12600	TadE family protein (TadE2)	12.9	593	11	44.8	3

The secretome and surface-attached proteomes revealed other interesting features. CAMP (Christie–Atkins–Much-Petersen) factor 1 was identified in all analyzed four type II strains with high coverage (ca. 60%) and highest identification scores, indicative of its prevalence. CAMP factor 2 was also detected with high scores (Supplementary Table S1).

### Presence of Cell Appendages in Plasmid-Positive *C. acnes* subsp. *defendens* Strains

**FIGURE 4:**
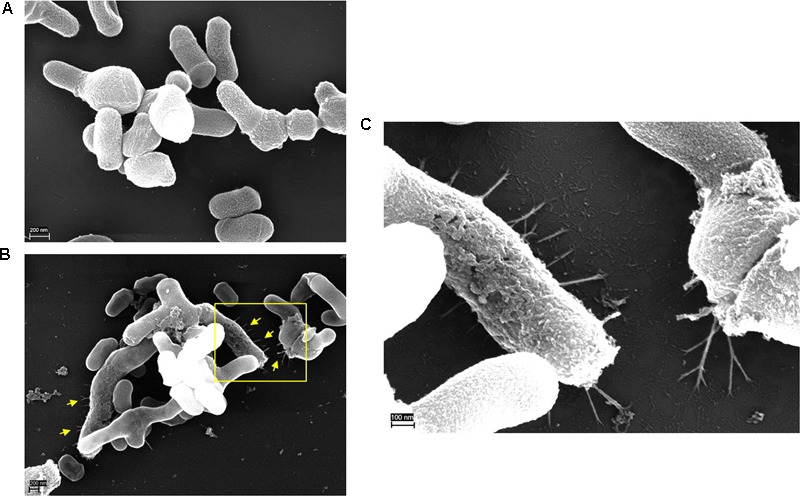
Scanning electron microscopy of plasmid-negative and -positive *C. acnes* cells. **(A)** Plasmid-negative strain 11-88; **(B)** plasmid-positive strain 11-79; **(C)** zoom-in **(B)**. Note various cell appendages (yellow arrows). Scale bars are shown on each image.

Next, we applied scanning EM to visualize putative cell appendages of type II *C. acnes*. Representative pictures show the distinct cell morphology of type II *C. acnes* with rather short, pleomorphic rods (**Figure 4**). The plasmid-negative *C. acnes* strain 11-88 does not show cell appendages (**Figure 4A**). In contrast, plasmid-positive strains have appendages in various lengths and sizes that might represent pili (**Figures 4B,C**). The diameter of these appendages is approximately 7 to 8 nm (**Supplementary Figure S2**), thus similar to pili of other bacteria. However, this EM analysis cannot unambiguously identify these appendages as Flp pili. A more specific approach, e.g., immunogold EM, targeting the Flp pilin with a specific antibody would be necessary.

### Plasmid Harbors Signatures for Its Conjugative Transfer

We conducted a reannotation of all ORFs of the plasmid; a locus of genes upstream of the *tad* locus was identified that encodes a variety of functions related to bacterial conjugation (**Figure 3**). These include a protein with a FtsK domain, similar to DNA translocases, such as TraB of *Streptomyces* sp.; TraB was shown to be crucial for the direct transfer from plasmid-carrying donor to the recipient (Thoma and Muth, 2016). Another protein encodes an ATPase of the VirB4 family; similar proteins are known conjugative transfer proteins (Rabel et al., 2003). A further protein shares similarity with TcpC, that is required for efficient conjugative transfer of the plasmid pCW3 from *Clostridium perfringens* (Porter et al., 2012).

In this respect, Blast searches revealed a striking similarity of the plasmid with a contig of a clostridial genome: a 40 kb contig (ABCB02000007) of the genome of *Clostridium leptum* strain DSM 753 (ATCC 29065), sequenced in 2007 as a reference genome for the NIH Human Microbiome Project. This contig is 99% identical on nucleotide level with plasmid p11-79 (nucleotide position 7884 to 45859) of a type II strain. This could either indicate a very recent interspecies conjugative plasmid transfer from *C. acnes* to *Cl. leptum* or it could represent a (sequencing) contamination. In order to investigate this, we re-sequenced *Cl. leptum* strain DSM 753 (ATCC 29065). We found that the newly sequenced genome (3,196,486 bp in 47 contigs) was about 73 kb smaller than the previously obtained genome (3,270,109 bp in 22 contigs). The new sequence did not contain any contigs with similarity to *C. acnes* plasmids. The G+C content of the *Cl. leptum* genome was 50%, thus substantially different from the G+C content of the *C. acnes* plasmid (63%). Taken together, we conclude that the most likely explanation for the presence of a *C. acnes* plasmid-like contig in the previously sequenced genome of *Cl. leptum* strain DSM 753 is a contamination. In fact, in addition to the 40 kb plasmid-like contig ABCB02000007 other contigs exist in the previous *Cl. leptum* DSM 753 genome submission that are identical to *C. acnes*. For example, contigs ABCB02000001 and ABCB02000002 are identical to chromosomal regions of *C. acnes* type II.

### Phylogenomics of Type II Strains and CRISPR/cas Loci

**FIGURE 5:**
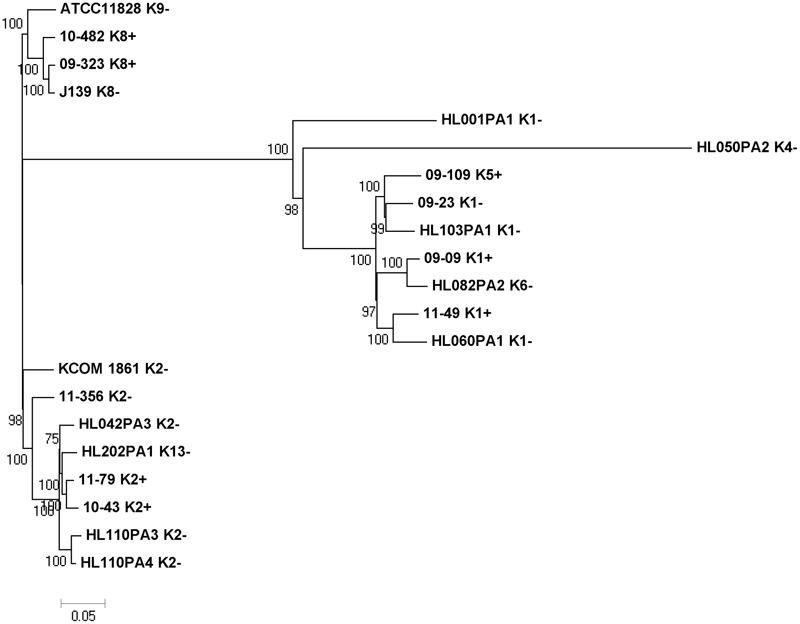
Phylogenomic comparison of strains of *C. acnes* subsp. *defendens.* The genomes of all nine newly sequenced type II strains and the available 12 type II genomes deposited in public databases (GenBank) were compared using the CSI phylogeny program. The phylogenetic analysis is based on the concatenated alignment of high quality SNPs. The SLST type (K type) of each strain is given after the strain name. Three main clades are identified, constituting of K8/K9, K2/K13 and K1/K4/K5/K6 strains, respectively.

Genomic information about type II strains is limited, but previous analysis based on a few genomes indicated higher genomic diversity of type II strains compared to type I strains (Scholz et al., 2016). The SLST scheme can currently separate 21 different sequence types (“K types”) among type II strains (Scholz et al., 2014). The newly sequenced type II strains belonged to the four K types K1, K2, K5, and K8. Comparison of whole genomes of all currently available type II strains revealed their heterogeneity (**Figure 5**). However, three main clusters can be distinguished, composed of strains of the K types (i) K8 and K9, (ii) K2 and K13 and (iii) K1, K4, K5, and K6. Plasmid-positive and -negative strains are mixed in all the three clusters.

One of the peculiarities of type II strains is the presence of a CRISPR/cas system; it was reported to be restricted to type II strains (Brüggemann et al., 2012). In agreement, all here sequenced type II strains possess a CRISPR/cas gene cluster. It was predicted that the CRISPR/Cas system would protect against the acquisition of the linear plasmid, since CRISPR/cas spacers were found that target sequences of the plasmid (Brüggemann et al., 2012; Tomida et al., 2013). However, we here found that plasmids are widespread in CRISPR/cas-positive type II strains. Only relatively few CRISPR/cas spacers, between one and six, were found in the newly sequenced genomes (Supplementary Table S2). Most spacers targeted various phages. Two strains (09-23 and 11-49) harbored spacers that targeted plasmid regions; one of these was plasmid-positive, indicating that the CRISPR/cas system in this strain is not functional or not sufficient to provide protection against plasmid acquisition. In agreement, the lack of protection against phage infection in some CRISPR/cas-positive type II strains was recently reported (Liu et al., 2015).

## Discussion

Phylogenetic analyses of *C. acnes* strains isolated from healthy skin and infectious sites, respectively, have suggested that specific phylotypes of the species may be associated with different pathological conditions. In the present study we investigated type II strains of *C. acnes*, the predominant type in a Swedish cohort of 181 isolates of *C. acnes* obtained from men with and without prostate cancer (Davidsson et al., 2016). We report the presence of a large plasmid in 20% of all investigated strains. This is to our knowledge the first report of the presence of plasmids in *C. acnes* isolates obtained from prostate tissue. Furthermore, the presence of the plasmid was more common in type II strains compared to type I strains in men with prostate cancer; however, more prostatic *C. acnes* isolates need to be analyzed in the future to confirm this finding.

A similar plasmid, described as being linear, was identified in type IA strains associated with acne, namely in strains of CC3 (Brüggemann et al., 2012; Kasimatis et al., 2013). In a large genome-based study on *C. acnes* strains isolated from acne-affected skin, it was shown that the majority of type I strains of the two acne-associated ribotypes 4 and 5 [largely equivalent with CC3 (type IA), and, in addition, strains of type IC] were plasmid-positive (Fitz-Gibbon et al., 2013; Tomida et al., 2013). Thus, it was believed that the presence of the plasmid is mainly a feature of acne-associated type I strains.

Like plasmids from type I strains, all newly sequenced plasmids contained a *tad* locus. The *tad* locus has been identified in many bacteria; it encodes the production adhesive Flp pili, also called type IVb pili or Tad pili (Tomich et al., 2007). Functional studies in *Actinobacillus actinomycetemcomitans* showed a role of Flp pili in adherence, and biofilm formation on surfaces (Kachlany et al., 2000). In addition, in a rat model the *tad* locus was found to be essential for colonization and persistency in the oral cavity (Schreiner et al., 2003). Similar functions of the Flp pili have been found for other bacteria. For instance, the *tad* locus in *Bifidobacterium breve* has been shown to be essential for efficient murine gut colonization (O’Connell Motherway et al., 2011). However, in *Micrococcus luteus* another role of the *tad* locus has recently been described (Angelov et al., 2015): whereas no role in adherence, cell aggregation or microcolony morphology could be detected, the locus was required for genetic transformation. Interestingly, other DNA transfer and, in particular, conjugation-related functions are encoded on the plasmid, such as a FtsK-like DNA translocase and proteins similar to VirB4 and TcpC; the corresponding homologs from *Streptomyces* sp., and *Clostridium perfringens*, respectively, are essential for conjugation (Rabel et al., 2003; Porter et al., 2012; Thoma and Muth, 2016). Thus, taken these aspects together, it is possible that the plasmid is actually a conjugative plasmid. Many conjugative plasmids in Gram-positive bacteria have been identified; at least two different strategies for horizontal plasmid transfer in Gram-positive bacteria are employed, i.e., transfer of single-stranded plasmid DNA via a type IV secretion system, and the macromolecular DNA translocation complex in streptomycetes that transports double-stranded DNA from donor to recipient cells (Goessweiner-Mohr et al., 2014; Thoma et al., 2015). The large plasmid of *C. acnes* encodes components of both systems. In this respect, it is unclear if the Flp pili are functionally linked to the putative conjugation apparatus.

Our study showed first evidence that plasmid-borne genes are functional in *C. acnes*. Four plasmid-encoded proteins were identified to be secreted and/or surface-exposed, including the putative pilin subunit TadE2. The proteomic data was supported by EM that revealed cell appendages protruding from plasmid-positive type II strains. Putative Flp pili-producing type II strains may be of interest concerning *C. acnes’* role in prostate cancer development, since *C. acnes* type II seems to be the most common type in prostatic specimens obtained from men with prostate cancer (Davidsson et al., 2016). It can be hypothesized that Flp pili-producing *C. acnes* type II would be more prone to colonize the prostate and trigger a sustained inflammation and thereby have a role in infection-induced prostate cancer. So far, nothing is known about the contribution of the plasmid of type II strains to their virulence. One study has compared a plasmid-positive with a plasmid-negative type IA strain to determine if they induce different immunological responses; it was shown that the plasmid-positive *C. acnes* type IA strain induced higher levels of IFN-γ in peripheral blood mononuclear cells (Yu et al., 2016). However, also plasmid-negative strains elicited a proinflammatory response.

Two *C. acnes* factors, CAMP factors and dermatan-sulfate adhesins (DsA1 and DsA2), are often mentioned with regard to their possible involvement in the proinflammatory activity of *C. acnes* (Lodes et al., 2006; Liu et al., 2011; McDowell et al., 2011, 2013; Nakatsuji et al., 2011; Lheure et al., 2016; Grange et al., 2017). We could show here that type II strains produce high levels of CAMP factors 1 and 2; the proteins were found with a high coverage, indicative of their abundancy. This is interesting, given the recent discovery that CAMP factor 1 of *C. acnes* is a ligand of Toll-like receptor (TLR) 2 (Lheure et al., 2016). The authors showed that purified CAMP factor 1 induces the production of the pro-inflammatory chemokine IL-8, and CAMP1-TLR2 binding appeared to be strong in type IB and II strains, which triggered the production of large amounts of IL-8 in keratinocytes, in contrast to most type IA strains that triggered low levels of IL-8. This would indicate that type II strains have an enhanced ability to induce this chemokine in exposed keratinocytes. In prostate cells this might be different, given the reported host cell tropism of *C. acnes* (Mak et al., 2012), but at present not much is known about the response of prostatic cells to type II *C. acnes*.

Dermatan-sulfate adhesins DsA1 and DsA2 of *C. acnes* have been partially characterized (Lodes et al., 2006; McDowell et al., 2011; Grange et al., 2017). They were found to be abundant in the secreted as well as surface-exposed fraction of *C. acnes* (Holland et al., 2010; Yu et al., 2016); moreover, they have been detected in human sebaceous follicles (Bek-Thomsen et al., 2014). A recent study has shown that DsA1 is highly glycosylated and recognizes human fibrinogen; thus, it is considered to be a MSCRAMM (microbial surface component recognizing adhesive matrix molecules) (Grange et al., 2017). The DsA1 protein sequence contains a repetitive motif of proline and threonine (PT repeats); the authors suggested that theses PT repeats could be responsible for fibrinogen-binding. Here, we could not identify DsA1 nor DsA2 among the secreted or surface-exposed factors of the tested type II strains, which is in agreement with previous findings showing that DsA1 is produced by *C*. *acnes* types IA and IC, but not by types IB, II, and III (Lodes et al., 2006; Holland et al., 2010; McDowell et al., 2011; Scholz et al., 2016). However, we could detect another PT repeat protein; this protein is encoded on a type II-specific insertion on the plasmid, thus is uniquely found in plasmid-positive type II strains. It needs to be investigated if this surface protein has dermatan-sulfate- and/or fibrinogen-binding properties.

This study provides genome sequences of nine type II strains, thus should help obtain new insight in *C. acnes* type II. Recently, reclassifications of cutaneous propionibacterial species were proposed: the species *P. acnes* was changed to *Cutibacterium acnes* (Scholz and Kilian, 2016). In addition, type II strains of *C. acnes* (*P. acnes*) were reclassified to *C. acnes* (*P. acnes*) subsp. *defendens* (McDowell et al., 2016). The name refers to the presence of a CRISPR/Cas system that is absent in all other *C. acnes* types. This was also confirmed in our study, showing that all investigated prostatic type II strains contained the CRISPR/cas system. We and others could show that the morphology of cells of *C. acnes* subsp. *defendens* are pleomorphic and shorter compared to type I cells (McDowell et al., 2016). This would also explain why certain studies detected coccoid instead of rod-shaped *C. acnes* in prostatic specimens (Bae et al., 2014; Kakegawa et al., 2017).

The present study provides detailed insight in prostatic *C. acnes* strains. To further investigate the biological plausibility for specific (i.e., plasmid-positive) *C. acnes* strains to be a contributing factor in prostate cancer development, future experiments have to be performed. First, the presence of Flp pili needs to be verified by more specific approaches, e.g., immunogold EM, targeting the Flp pilin. To study the putative role of Flp pili in *C. acnes*, a knockout mutagenesis approach should be applied that targets the *tad* gene locus. A first attempt to create a knock-out mutant in a type II strain failed. To our knowledge, knockout mutations have successfully been created only in one specific *C. acnes* strain so far (strain KPA171202, type IB, plasmid-negative) (Sörensen et al., 2010). Furthermore, it needs to be shown in the future if Flp pili are important for adherence and colonization of prostatic tissues. Moreover, the impact of plasmid-positive *C. acnes* strains on (persistent) prostatic inflammation and the cellular fate needs to be investigated.

## Ethics Statement

This study was carried out in accordance with the recommendations of the Ethical Review Board in Uppsala-Örebro, Sweden. All subjects gave written informed consent in accordance with the Declaration of Helsinki. The protocol was approved by the Ethical Review Board in Uppsala-Örebro, Sweden (2008/293).

## Author Contributions

SD, JC, and HB conceived, designed and performed the experiments, analyzed data and wrote the manuscript. OA and S-OA performed the surgical operations. BS, PM, and NG performed the PCR experiments and analyzed the data. EB and AP performed the genome sequencing. MA-Z and VB performed sample preparation and electron microscopy. CS and SN performed sample preparation and mass spectrometric analysis.

## Conflict of Interest Statement

The authors declare that the research was conducted in the absence of any commercial or financial relationships that could be construed as a potential conflict of interest.

## Footnotes

1 http://www.bioinformatics.babraham.ac.uk/projects/fastqc/

2 http://www.usadellab.org/cms/?page=trimmomatic

3 http://www.sanger.ac.uk/science/tools/artemis-comparison-tool-act

4 http://www.sanger.ac.uk/science/tools/artemis-comparison-tool-act

5 https://cge.cbs.dtu.dk/services/CSIPhylogeny/

6 http://crispr.i2bc.paris-saclay.fr/Server/

## Supplementary Material

FIGURE S1 Possible pilin subunits of Flp pili of *C. acnes*. **(A)** Tad E1 (APS60_12595) contains a possible prepilin cleavage site (in bold). It is predicted to be a prepilin according to a search with the Pilfind server (http://signalfind.org/ pilfind.html). The predicted motif is underlined. Detection in a MS approach based on trypsin digestion is difficult due to the absence of arginine residues in the mature pilin; lysine residues can only be found in the terminal part of the C-terminus. **(B)** Alignment of TadE2 (APS60_12600), TadE3 (APS60_12605), and TadE4 (APS60_12610), showing a conserved N-terminus (in bold). The TadE/pfam07811-like domain is highlighted in yellow. **(C)** The identified peptides of TadE2 by MS analysis of the surface-exposed fraction of *C. acnes* (strains 11-79 and 09-9) are highlighted in red.

FIGURE S2 Electron microscopy of appendages of *C. acnes* and assessment of their sizes.

## Abbreviations

CAMP: Christie–Atkins–Much-Petersen
CC: clonal complex
EM: electron microscopy
Flp: fimbrial low-molecular weight protein
MLST: multi-locus sequence typing
MS: mass spectrometry
PT: proline-threonine
RCM: reinforced clostridial medium
SLST: single-locus sequence typing
tad: tight adherence
