# Supplementary material for: Prevalence of Flp Pili-Encoding Plasmids in Cutibacterium acnes Isolates Obtained from Prostatic Tissue
Source: Front Microbiol. 2017 Nov 16;8:2241. doi: 10.3389/fmicb.2017.02241 (PMC5696575; doi:10.3389/fmicb.2017.02241)
Supplement: FIGURE S1 — Possible pilin subunits of Flp pili of C. acnes. (A) Tad E1 (APS60_12595) contains a possible prepilin cleavage site (in bold). It is predicted to be a prepilin according to a search with the Pilfind server (http://signalfind.org/ pilfind.html). The predicted motif is underlined. Detection in a MS approach based on trypsin digestion is difficult due to the absence of arginine residues in the mature pilin; lysine residues can only be found in the terminal part of the C-terminus. (B) Alignment of TadE2 (APS60_12600), TadE3 (APS60_12605), and TadE4 (APS60_12610), showing a conserved N-terminus (in bold). The TadE/pfam07811-like domain is highlighted in yellow. (C) The identified peptides of TadE2 by MS analysis of the surface-exposed fraction of C. acnes (strains 11-79 and 09-9) are highlighted in red. [file Image_1.PDF]

A

TadE1 MMTDLDLAKKQITWLAAHLRSRIVDERGG**GSSTVET**LIWIGLVVATVVAVGAVVMAFIKS
KMPH

B

```
TadE2      MRSRFTTDQRGGGSASVGMLLLMPAIMLLAFGGIEVGMWCHAHQSTIAAAQS--------
TadE3      ----MRRSERGGASLSVEVLMWAPIALVIIGFVVGIGRMSMAQDAVSGAAGAAARAA---
TadE4      ----MARDERGGGSVSVWMLLMVPVILVMAGLVFDGSRQISATQAAQDAAVAASRAGTDA
               :  .:***.* ** :*:  *  :::    .  .    * ::.  ** :

TadE2      AAEAQRVVHPAPGSAQEAASQITSHGGVRDARVTVRDDGATVTVTVSGRAPSMLG-----
TadE3      --SLERDGQSAQSAAQQAASANLSSGGLACA-PSVSVDTSVFARPAGQA-GTVH-ATVTC
TadE4      AATPQLAGHDGAAVAVQAARQALSAAGVDG---SVQEDGSTITVTTSQSRPTVFLSAIGI
               :   : . . * :**    * .*:     :*  * :..:  ..    ::

TadE2      -------LHLPAVSSTASMPKERLS----
TadE3      VTSLGLGFGSRTVHATGSAPVDTYRERRG
TadE4      SQVRGHGQAHAQLVGPGERP---------
                   : . .. *
```

C

>TadE2
MRSRFTTDQRGGGSASVGMLLLMPAIMLLAFGGIEVGMWCHAHQSTIAAAQSAAEAQRVV
HPAPGSAQEAASQITSHGGVRDARVTVRDDGATVTVTVSGRAPSMLGLHLPAVSSTASMP
KERLS

Figure S1: Possible pilin subunits of Flp pili of *C. acnes*

A, Tad E1 (APS60_12595) contains a possible prepilin cleavage site (in bold). It is predicted to be a prepilin according to a search with the Pilfind server (http://signalfind.org/pilfind.html). The predicted motif is underlined. Detection in a MS approach based on trypsin digestion is difficult due to the absence of arginine residues in the mature pilin; lysine residues can only be found in the terminal part of the C-terminus. B, Alignment of TadE2 (APS60_12600), TadE3 (APS60_12605) and TadE4 (APS60_12610), showing a conserved N-terminus (in bold). The TadE/pfam07811-like domain is highlighted in yellow. C, The identified peptides of TadE2 by MS analysis of the surface-exposed fraction of *C. acnes* (strains 11-79 and 09-9) are highlighted in red.
