# Supplementary material for: Prevalence of Flp Pili-Encoding Plasmids in Cutibacterium acnes Isolates Obtained from Prostatic Tissue
Source: Front Microbiol. 2017 Nov 16;8:2241. doi: 10.3389/fmicb.2017.02241 (PMC5696575; doi:10.3389/fmicb.2017.02241)
Supplement: Supplementary file 4 [file Table_2.DOCX]

Table S2: Identified CRISPR/cas spacer sequences in the nine sequenced type II *C. acnes* genomes

| **strain** | **repeat** | **spacers** |
| --- | --- | --- |
| 11-356 | GTATTCCCCGCCTATGCGGGGGTGAGCCCG | AGGGCTACCACGTGGTCGATTTGGACTGTCG |
|  |  | GGCGCTCCACTCCCTCGCCCTGGCCACCAAC |
| 09-323 | GGGCTCACCCCCGCATAGGCGGGGAATAC | GTTGGTGGCCAGGGCGAGGGAGTGGAGCGCCT |
|  |  | CGACAGTCCAAATCGACCACGTGGTAGCCCTC |
| 11-79 | GGGCTCACCCCCGCATAGGCGGGGAATAC | GTTGGTGGCCAGGGCGAGGGAGTGGAGCGCCT |
|  |  | CGACAGTCCAAATCGACCACGTGGTAGCCCTC |
| 10-482 | TGGGCTCACCCCCGCATAGGCGGGGAATAC | GTTGGTGGCCAGGGCGAGGGAGTGGAGCGCC |
| 10-43 | GGGCTCACCCCCGCATAGGCGGGGAATAC | GTTGGTGGCCAGGGCGAGGGAGTGGAGCGCCT |
|  |  | CGACAGTCCAAATCGACCACGTGGTAGCCCTC |
|  |  | CGTCTGGGCAGGTGGACGATATTTTGGCGCGT |
|  |  | CACCCAATACACCCCATGAAACGCTCACAATT |
|  |  | CCTCACACAACACCGTGGCACCCTGAACCGTA |
|  |  | TCTGATCATGCAAGGCTTTCACATCAGCCACA |
| 09-09 | GGCTCACCCCCGCATAGGCGGGGAATAC | CCGAGAGGACTATGACGCTGATGACTGGTGTGA |
| 11-49 | GTATTCCCCGCCTATGCGGGGGTGAGCC | CGGGCCAGGGCAACGGGGCCGCATGCGACTCCG |
|  |  | CGCCTACCGTCAGCTGACTCACGCCTCCGCGTT |
|  |  | TCACACCAGTCATCAGCGTCATAGTCCTCTCGG |
| 09-23 | GTATTCCCCGCCTATGCGGGGGTGAGCC | GTGGCCGTACTCGCACGCCAACAAGGCAAAACC |
|  |  | CACCACTCGGGGTGGGACTGCCCAGTTTTATTG |
|  |  | TCACACCAGTCATCAGCGTCATAGTCCTCTCGG |
| 09-109 | GGCTCACCCCCGCATAGGCGGGGAATAC | CCGAGAGGACTATGACGCTGATGACTGGTGTGA |
